# Supplementary material for: Short communication: A comparison between two glucose measurement methods in beef steers during a glucose tolerance test
Source: PLoS One. 2022 Jul 19;17(7):e0271673. doi: 10.1371/journal.pone.0271673 (PMC9491837; doi:10.1371/journal.pone.0271673)
Supplement: Attachment — Submitted filename: Response to Reviewers PLOSone Glucose steers.docx [file pone.0271673.s002.docx]

**Reviewer #1: This manuscript aimed to evaluate the effectiveness of a hand-held glucometer in beef cattle The manuscript is within the scope of the Plos One and can be accepted for publication. However, this manuscript should be classified as a short communication.**

The authors have changed the title to reflect the reviewer’s comment.

**Reviewer #2:**

**Lines 56-59 - I really didn’t understand why you are pointing out these limitations and possible errors of the laboratory method as a justification for using the hand-held glucometer. Because you are using this method as a gold-standard method for your comparison and recommendation of the alternative method.**

The authors appreciate the reviewer’s comment. We agree, that the limitations of the laboratory method should not be pointed out since this is our gold standard method of comparison. However, the justification for the use of the hand-held glucometer is that it is much faster and cheaper to use compared with the laboratory method. We have changed the text in lines 56-57 to accommodate the reviewer’s point.

**Lines 60-62 - This sentence sounds very informal to a paper’s introduction. I believe that should be in a more appropriate place, such as discussion.**

The authors thank the reviewer for this recommendation. We have removed this sentence from the introduction and have added it to the concluding paragraph in our discussion (lines 242-244).

**Lines 68-70 - What is the real necessity to validate the same method for beef cattle? Why the results should be different for beef cattle when compared with dairy cattle? We are talking about the method.**

The authors appreciate the reviewer’s question. First, there is evidence that there are differences in both hormone and metabolite profiles when comparing beef cattle with dairy cattle. Furthermore, we refer to this published difference in the manuscript. Therefore, we feel that there may be differences in plasma glucose responses to a glucose tolerance test in beef cattle (i.e. peak plasma glucose concentration, baseline concentration, etc.). This is the rationale for validating the same method for beef steers, as there may be physiological differences that we are unaware of, and no one to our knowledge has validated this for beef cattle. We have added text in lines 70-74 to indicate this. Second, we suggest that a complete analysis of agreement between methods to measure glucose concentrations in blood, published with dairy cattle is not available. In particular the use of Lin’s concordance correlation to assess agreement. We have included this statistical method in our analysis. Third we suggest that replication of results between authors confers confidence in future users of the technique.

**Line 101 and line 104 – Is the range of reading from 20 to 300 or 20 to 500? There is confusion here.**

The authors thank the reviewer for pointing out this error in the text. The glucometer has a range of 20-500 mg/dL, and this has been changed from 300 to 500 in line 105.

**Lines 105-107 - This is not correct. This sample should be deleted from the database because you are not certain about the value.**

We thank the reviewer for this recommendation. However, we believe that this sample should remain in the dataset. The objective of this paper was to evaluate the agreeance between the two methods, and this sample is still a valid result of the glucometer based on the glucometer manual. We feel that removing this sample or any other potential samples that were greater than 500 mg/dL would skew the results and could give a false agreeance of the two methods as the greater concentrations were removed from the dataset (even though there was only the one result that was greater than 500 mg/dL in this paper). The Bland-Altman method takes into consideration the difference between the two methods’ measurement of the glucose concentration. If this point greatly differs from the laboratory method, the Bland-Altman analysis will capture this, therefore we feel that this data point should remain in the data set.

**Lines 118-120 - Why did you not test for normality?? You need to be certain about distribution because that could be biasing the results.**

The authors appreciate the reviewer’s comment. We did not test for normality of the dataset because the agreeance of the two methods was evaluated during a glucose tolerance test. Therefore, it is likely that the data will not be normal because of the skewed and elevated plasma glucose concentrations that occur during a glucose tolerance test. Additionally, the Bland-Altman method is assessing the difference between the two measurements in a pair. This is why we assessed normality of the residuals in SAS, as the t-test and the correlation that were completed both evaluate the differences between the two methods and to see if there was a systematic effect on the differences. This is further explained in the results section of the manuscript.

Additionally, Bland and Altman (2010) state “Such differences are likely to follow a normal distribution because we have removed a lot of the variation between subjects and are left with the measurement error. The measurements themselves do not have to follow a normal distribution, and often they will not.”

**Lines 120-123 – In a general way, the information concerning statistical analysis and methods for assessing adequacy were poorly described in this section. Some explanations about these methods are dispersed over the results and discussion, which is not the best place for that. I suggest that authors should be providing these details in the current section.**

The authors thank the reviewer for this comment. We agree with the reviewer and have moved much of the statistical analysis explanation that was provided in both the results and discussion to the statistical analysis section of the Materials and Methods. We have also added some additional information on the statistical analysis procedure that was used following the recommendations of Petrie and Watson (2013) in lines 126-144.

**Line 132 - Why did you not include regression to test the adequacy of your new method?**

We appreciate the reviewer’s inquiry. Based on Bland and Altman (1983), linear regression is an incorrect technique in method comparison studies. The authors state “Often the slope of the least squares regression line is tested against zero. This is equivalent to testing the correlation coefficient against zero”. The correlation coefficient is not a measure of agreement, it is a measure of association, and we agree with Bland and Altman (1983) that linear regression similarly looks at only association, not agreement.

**Lines 137-139 - I am really not sure about the confidence to test transformed variables. It is proved that variables transformation to follow Gaussian distribution, do not represent a good estimate of the real value when a back-transformation is applied. I believe that is a similar case. The funnel shape is also present in the transformed data, but with lower intensity. The authors should think about that.**

The authors appreciate the reviewer’s comment. However, based on the Bland and Altman (1986) methodology, it is acceptable to transform the data. The authors discuss that if the differences are proportional to the mean, a logarithmic transformation can be completed, and you can then apply the analysis to the log-transformed data.

**Lines 142-1444 - It is clear that there is a bias over 300 mg/dL (Figures 1 and 2) and I believe that should be analyzed and reported in the manuscript.**

The authors thank the reviewer for this recommendation. We have, however, discussed this in the discussion in lines 228-243.

**Lines 160-174 - This entire paragraph is closer to a material and methods than a discussion.**

The authors agree with the reviewer and have moved/amended this text to fit into the materials and methods under the statistical analysis section.

**Lines 215-218 - Which is the necessity to repeat this text?**

The authors appreciate the reviewer’s question. We have shortened the text in lines 220-223 to avoid repeating the materials and methods, however, we wish to leave the sampling timeline in the discussion as we are directly comparing our sampling timeline to the Wittrock et al. (2013) sampling timeline and do not want readers to have to go back to the materials and methods to remember what our timeline was.
